# Association between iron accumulation in the dorsal striatum and compulsive drinking in alcohol use disorder

**DOI:** 10.1007/s00213-022-06301-7

**Published:** 2022-12-29

**Authors:** Haoye Tan, Simon Hubertus, Sebastian Thomas, Alycia M. Lee, Sarah Gerhardt, Martin Fungisai Gerchen, Wolfgang H. Sommer, Falk Kiefer, Lothar Schad, Sabine Vollstädt-Klein

**Affiliations:** 1 Department of Addictive Behaviour and Addiction Medicine, Central Institute of Mental Health, Medical Faculty of Mannheim, Heidelberg University, 68159 Mannheim, Germany; 2 Computer Assisted Clinical Medicine, Medical Faculty of Mannheim, Heidelberg University, 68159 Mannheim, Germany; 3 Department of Clinical Psychology, Central Institute of Mental Health, Medical Faculty of Mannheim, Heidelberg University, 68159 Mannheim, Germany; 4 Bernstein Center for Computational Neuroscience Heidelberg/Mannheim, 68159 Mannheim, Germany; 5 Department of Psychology, Heidelberg University, 69117 Heidelberg, Germany; 6 Institute of Psychopharmacology, Central Institute of Mental Health, Heidelberg University, 68159 Mannheim, Germany; 7 Bethania Hospital for Psychiatry, Psychosomatics, and Psychotherapy, Greifswald, Germany; 8 Mannheim Center for Translational Neurosciences (MCTN), Medical Faculty of Mannheim, Heidelberg University, 68159 Mannheim, Germany; 9 Feuerlein Center on Translational Addiction Medicine, Heidelberg University, 69117 Heidelberg, Germany

**Keywords:** Brain iron, Alcohol use disorder, Quantitative susceptibility mapping, Compulsive drinking, Dorsal striatum

## Abstract

**Rationale:**

Brain iron accumulation has been observed in neuropsychiatric disorders and shown to be related to neurodegeneration.

**Objectives:**

In this study, we used quantitative susceptibility mapping (QSM), an emerging MRI technique developed for quantifying tissue magnetic susceptibility, to examine brain iron accumulation in individuals with alcohol use disorder (AUD) and its relation to compulsive drinking.

**Methods:**

Based on our previous projects, QSM was performed as a secondary analysis with gradient echo sequence images, in 186 individuals with AUD and 274 healthy participants. Whole-brain susceptibility values were calculated with morphology-enabled dipole inversion and referenced to the cerebrospinal fluid. Then, the susceptibility maps were compared between AUD individuals and healthy participants. The relationship between drinking patterns and susceptibility was explored.

**Results:**

Whole-brain analyses showed that the susceptibility in the dorsal striatum (putamen and caudate) among AUD individuals was higher than healthy participants and was positively related to the Obsessive Compulsive Drinking Scale (OCDS) scores and the amount of drinking in the past three months.

**Conclusions:**

Increased susceptibility suggests higher iron accumulation in the dorsal striatum in AUD. This surrogate for the brain iron level was linearly associated with the compulsive drinking pattern and the recent amount of drinking, which provides us a new clinical perspective in relation to brain iron accumulation, and also might indicate an association of AUD with neuroinflammation as a consequence of brain iron accumulation. The iron accumulation in the striatum is further relevant for functional imaging studies in AUD by potentially producing signal dropout and artefacts in fMRI images.

**Supplementary Information:**

The online version contains supplementary material available at 10.1007/s00213-022-06301-7.

## Introduction

As a chronic relapsing disease, alcohol use disorder (AUD) represents one of the world’s most significant addiction problems and has a large impact on global public health. It is characterized by recurrent compulsive alcohol use despite significant alcohol-related behavioral, cognitive, physiological, and social problems. The Diagnostic and Statistical Manual of Mental Disorders fifth version (DSM-5) criteria of substance use disorder also emphasizes the compulsive quality as a central aspect of addiction (American Psychiatric Association 2013).

The underlying neurobiological mechanism of compulsive consumption is currently still not fully understood. Converging evidence suggests the dorsal striatum to be critical in compulsive drug seeking. In animal studies, a large increase in dopamine levels was observed in the dorsal striatum in long-term cocaine use (Ito et al. 2002), and when inactivating the dorsolateral striatum, the habitual behavior was reduced (Vanderschuren et al. 2005). In addition to the deep gray matter of the striatum, a circuit involving the frontal cortex is suggested to be important for the development of compulsivity. In human imaging studies, our previous results from cue–reactivity tasks indicated that the cue-induced activation of the ventral striatum in social drinkers is higher than in heavy drinkers, while in heavy drinkers it was higher in dorsal striatum (Vollstädt-Klein et al. 2010). This suggested that the dorsal striatum became the dominant region in compulsive alcohol use. In 2013, Sjoerds et al. also found a dysfunction of the anterior putamen in alcohol-dependent patients using an instrumental learning task, which was related to habit control (Sjoerds et al. 2013). From an anatomical perspective, studies using structural MRI have indicated that the basal ganglia were affected in alcohol users, including the caudate, putamen, and nucleus accumbens (Fritz et al. 2022).

Brain iron concentration has emerged as a potentially contributing factor to psychiatric disorders. In 2017 (Juhás et al. 2017), brain iron accumulation in the deep gray matter of AUD patients was ascertained from resting-state functional MRI (fMRI) signal, by combining multi-channel complex phase signal in raw fMRI data using an adaptive method. Patients exhibited higher iron levels in the basal ganglia regions including the caudate nucleus, putamen, globus pallidus, and dentate nucleus compared to healthy subjects. Recently, a study based on UK Biobank also found moderate alcohol consumption was associated with higher iron in the putamen, caudate, and substantia nigra (Topiwala et al. 2022b). Previous research found brain iron levels to not only be associated with aging and neurodegeneration (Möller et al. 2019) but also with some psychiatric disorders, such as mood disorders and schizophrenia (Necus et al. 2019; Yao et al. 2017), whereby the role of concomitant alcohol use remains unclear in these studies. With regard to substance use disorder (SUD), iron accumulation was observed in the globus pallidus of cocaine users, which strongly correlated with the overall duration of cocaine use (Ersche et al. 2017). Similarly, accumulation of iron in the globus pallidus and substantia nigra was found in methamphetamine-exposed animals (Melega et al. 2007). These findings showed that in SUD the basal ganglia exhibited an increased iron concentration. The mechanism of this restricted pattern of iron accumulation in the brain are not well understood. The profound effect of alcohol on systemic iron storage is well established (Duane et al. 1992; Whitfield et al. 2001), and animal studies suggest an involvement of dopamine signaling in brain iron metabolism (Ben-Shachar et al. 1993; Ben-Shachar and Youdim 1990). Interestingly, the basal ganglia, especially the ventral and dorsal striatum, as mentioned above, are also at the core of the shift from hedonic to compulsive consumption.

Quantitative susceptibility mapping (QSM) is an emerging MRI technique. It calculates the tissue frequency shift using phase information at different echo times from gradient echo images and then reconstructs the susceptibility maps (Haacke et al. 2015; Kurz et al. 2021; Möller et al. 2019; Wang and Liu 2015). Studies have shown that in gray matter structures there is a strong linear correlation between chemically determined iron concentration and bulk magnetic susceptibility (Langkammer et al. 2012). This method has been extensively validated to be able to identify altered deep grey matter iron in normal aging as well as in many neurological disorders (Deistung et al. 2017; Haacke et al. 2015; Wang and Liu 2015).

Here, we applied QSM with gradient multi-echo imaging collected by and compiled from several previously conducted fMRI studies to compare brain iron levels in individuals with AUD and healthy participants. We hypothesized that AUD individuals show increased accumulation of brain iron especially in the basal ganglia and that the concentration of brain iron relates to compulsive drinking.

## Methods

### Participants

This study was based on previous projects (Bach et al. 2021; Bach et al. 2019; De Santis et al. 2019; Gerchen et al. 2021; Hansson et al. 2018; Karl et al. 2021; Vollstädt-Klein et al. 2020) in AUD conducted in our lab (Supplementary Table 1), which were all designed with similar inclusion criteria and used the same gradient echo sequence (GRE). Data were collected for 186 AUD individuals (DSM-IV or DSM-5 criteria, see supplementary material) and 274 healthy participants recruited between 2011 and 2019 at the Central Institute of Mental Health, Mannheim, Germany. The demographic and clinical overview of the participants is summarized in Table 1. AUD individuals did not use other substances except nicotine, which was verified by a urine drug screen (nal von minden GmbH Drug-Screen® Diptest, Version 1.0). The healthy participants had no history of alcohol or drug addiction or any current psychiatric disorder. Participants in both groups were excluded if they had any history of serious medical (including psychiatric or neurological) complications, brain injury, use of psychotropic medications (other than during the detoxification process), or did not meet magnetic resonance safety criteria for our imaging facility, for example because of metal implants or pregnancy.

**Table 1:** Group characteristics of all participants (*N* = 460)

	AUD individuals	Healthy controls	Statistics (*T*/*χ*^2^ value)	*df*	*P* value
*N*	186	274			
Age (years)	48.3 ± 10.8	37.5 ± 15.3	8.337	458	< 0.001
Sex (female)	34, 18.3%	66, 24.2%	1.924	1	0.165
Duration of drinking (years)	19.8±12.9	-	-	-	-
Cumulative amount of alcohol (gram in the last 90 days)^a^	15332.2 ± 13752.9	1092.1 ± 3967.3	9.227	206	< 0.001
Current smoke (yes)	115, 67.6%	30, 13.8%	116.206	1	< 0.001
ADS score	11.8 ± 8.0	2.5 ± 3.5	9.271	130	< 0.001
AUQ score	13.5 ± 6.5	10.3 ± 3.6	4.503	218	< 0.001
OCDS global^b^	14.8 ± 7.5	2.8 ± 3.9	15.619	251	< 0.001

Abbreviations: *ADS*, Alcohol Dependence Scale; *AUQ*, Alcohol Urge Questionnaire; *OCDS*, Obsessive Compulsive Drinking Scale (OCDS)

^a^Based on FORM90

^b^Calculation rules of OCDS based on Mann et. al. (Mann and Ackermann 2000)

Before taking part in the scanning procedure, participants completed the following questionnaires: Form90 (Scheurich et al. 2005), the Alcohol Dependence Scale (ADS, (Kivlahan et al. 1989)), the Alcohol Urge Questionnaire (AUQ, (Bohn et al. 1995)), and the Obsessive Compulsive Drinking Scale (OCDS, (Anton et al. 1995; Mann and Ackermann 2000)). Form90 retrospectively recorded the amount of alcohol drunk everyday and calculated the cumulative amount in the past 90 days. With Form90, the amount of daily alcohol consumption was assessed, and the cumulative amount in the past 90 days was calculated. All participants provided informed written consent according to the declaration of Helsinki, and all projects in this study were approved by the ethics committee of the University of Heidelberg.

### MRI acquisition

Neuroimaging data was acquired using a Siemens 3 Tesla whole-body tomograph (MAGNETOM Trio, TIM technology, Siemens, Erlangen, Germany) with a 12-channel head coil. A multislice 2D-GRE was used for the QSM analysis: TR = 358 ms; TE_1_ = 5.19 ms, and TE_2_ = 7.65 ms; matrix size = 64 × 64 × 42; voxel size = 3 × 3 × 3 mm^3^; and flip angle = 60°. This sequence was originally implemented as a sequence for fieldmap correction of fMRI data to control for distortions of the functional images in the previous projects.

### Quantitative susceptibility mapping (QSM)

The GRE raw data were reconstructed manually by using a sum-of-squares approach for the magnitude and exponential addition for the phase after referencing the phase of each channel to the first echo. QSM reconstruction was done with the MEDI toolbox from Cornell MRI Research Lab (de Rochefort et al. 2010; Liu et al. 2011), which included procedures of fitting the complex MRI data, phase unwrapping with a region growth approach, brain mask generation with morphological operators and 5 mm erosion of the boundary, and background field removal by solving the Laplacian boundary value (Sun and Wilman 2014; Zhou et al. 2014). Furthermore, field inversion with MEDI used a weighting factor of 1000, which was based on the parameter optimization (from 10^−3^ to 10^6^) with 10% random sub-sampling (for detailed methodological description see (Hubertus et al. 2019a, b)), and the susceptibility maps were also referenced to the averaged susceptibility in the cerebrospinal fluid (CSF). The CSF-referenced susceptibility values were relative values without units.

### Data analysis

The CSF-referenced susceptibility maps were then normalized using SPM12 (Wellcome Centre for Human Neuroimaging, London, UK, https://www.fil.ion.ucl.ac.uk/spm/) to SPM12 TPM MNI template for statistical comparison. Whole-brain susceptibility values for each subject were included in a one-tailed *t*-test to find brain regions with differences in QSM intensity between the groups of AUD and healthy participants. Age and current smoke status were added as covariates of no interest. Although we had specific hypotheses for the basal ganglia, we conducted whole brain analyses to also exploratory look at other brain regions. A voxel-wise-threshold of *P* < 0.001 in combination with a cluster-extent threshold determined with random field theory in SPM12 was used for a corresponding cluster-level family-wise error (FWE) significance threshold of *P* < 0.05. We then generated a region of interest (ROI) using the significant voxels of the group comparison and averaged the susceptibility values in this ROI. A linear partial correlation controlling for age and smoke status was conducted between the mean susceptibility within the ROI and psychometric variables using SPSS (Statistical Package of the Social Sciences, version 25; SPSS Inc., Chicago, IL, USA). Correlation analyses were done to all participants, because in a group of healthy participants, there were also light to moderate drinkers, which could bring more information on linear relations between susceptibility and psychometrics. Psychometric data included the sum of ADS score, AUQ score, and the OCDS global score, according to the calculation rules from a previous study (Mann and Ackermann 2000).

## Results

### Whole-brain susceptibility in AUD and healthy participants

**Fig. 1:**
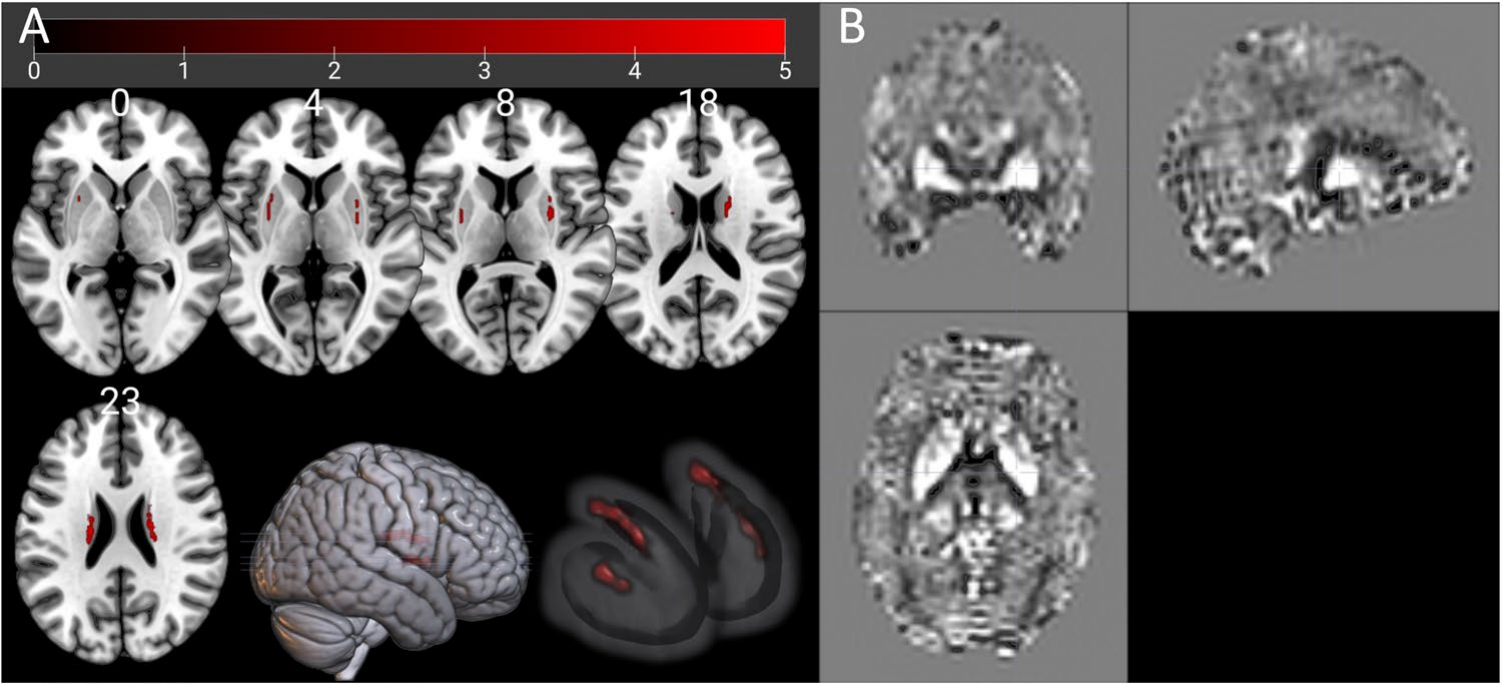
Brain regions with iron accumulation. (**A**) Whole-brain two-sample *t*-test of susceptibility between AUD individuals and healthy participants (combined voxel-wise (*P* < 0.001) and extent threshold FWEc = 29 voxels, corresponding to cluster-pFWE < 0.05). (**B**) An exemplary susceptibility map from an AUD individual

To examine the brain iron level, we voxel-wise compared the whole-brain susceptibility in AUD individuals with the healthy participants. AUD individuals showed increased CSF-referenced susceptibility in the bilateral putamen and caudate (Table 2 and Fig. 1). This revealed higher iron accumulation in the dorsal striatum of AUD individuals.

**Table 2:** Brain areas with increased susceptibility in AUD individuals compared to healthy controls (*n* = 460 subjects, combined voxel-wise (*P* < 0.001) and FWEc = 29 voxels, corresponding to cluster-pFWE < 0.05)

Side	Brain regions	Percentages in cluster	Cluster size	MNI coordinates (*x*, *y*, and *z*)	*t*_max_
Left	Caudate	86.4%^a^	44	− 20 − 20 22	5.1025
Right	Caudate	83.6%^b^	67	18 2 20	5.0614
Right	Putamen	100%	37	28 6 6	4.6831
Left	Putamen	100%	29	− 28 − 2 6	4.0510

^a, b^The rest voxels of these two clusters were unlabeled in AAL-Atlas

### Correlation of susceptibility and psychometrics

**Fig. 2:**
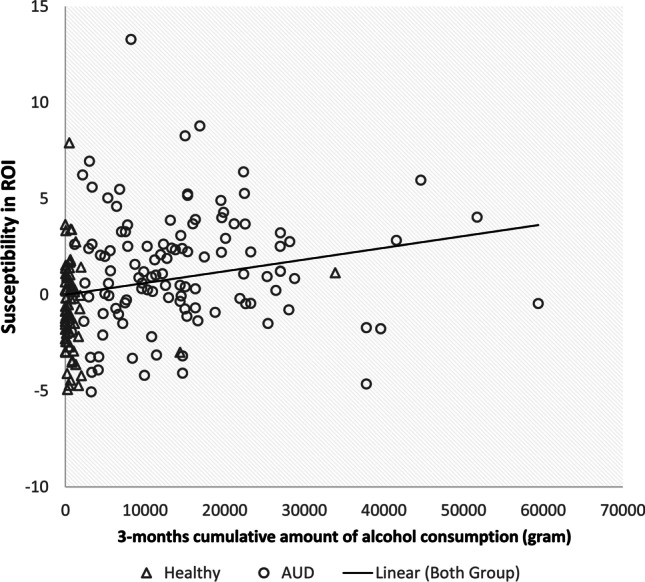
Correlation of the cumulative amount of drinking (alcohol in gram, in the last 90 days period) and susceptibility, controlling for age and smoking status

**Fig. 3:**
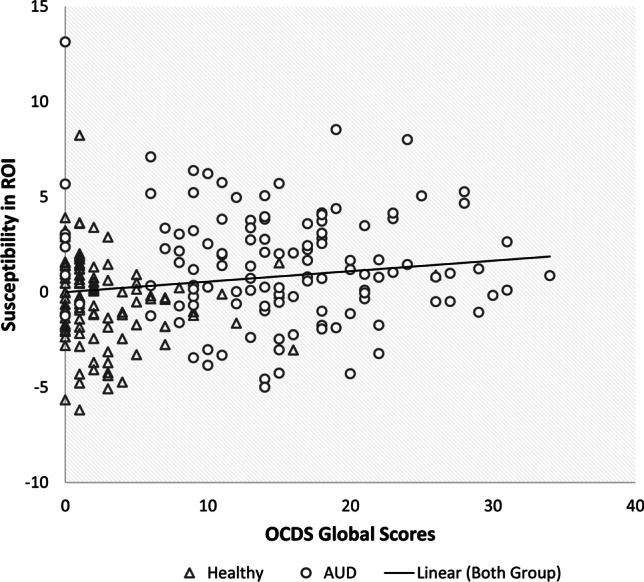
Correlation of OCDS scores and susceptibility, controlling for age and smoking status

To explore whether the increased susceptibility in the dorsal striatum is related to the pattern of alcohol consumption, further correlation analyses were conducted. The mean susceptibility in the ROI of all 177 voxels in four clusters based on the results of whole-brain analysis was positively linearly correlated to the cumulative amount of alcohol consumption in the past three months, controlling for age and smoke status (Table 3 and Fig. 2). Furthermore, the ROI susceptibility was also significantly correlated to OCDS global scores (Table 3 and Fig. 3). There was no significant correlation with ADS and AUQ observed (sFigure 1 and sFigure 2), and the linear correlations were not significant within the AUD group.

**Table 3:** Correlation of mean ROI susceptibility and psychometric variables in all participants

Controlled age and smoke status	All participants			AUD group		
	Coefficient	*df*	*P*	coefficient	*df*	*P*
Cumulative amount of drinking (gram in the last 90 days)	0.201	185	0.006	0.068	110	0.478
Global score of OCDS ^a^	0.146	225	0.028	-0.072	118	0.434
Sum of ADS	0.139	120	0.127	0.190	40	0.228
Sum of AUQ	0.118	201	0.093	0.097	104	0.320

Abbreviations: *ADS*, Alcohol Dependence Scale; *AUQ*, Alcohol Urge Questionnaire; Obsessive Compulsive Drinking Scale (OCDS)

^a^Calculation rules of OCDS based on Mann et al. (Mann and Ackermann 2000)

## Discussion

The most salient message of the current study is that AUD patients show increased iron accumulation in the dorsal striatum and that iron levels are associated with the measure of drinking pattern. Specifically, AUD subjects had bilaterally increased magnetic susceptibility in the dorsal striatum when compared to healthy participants. Importantly, this iron accumulation was strongly and positively correlated to the alcohol exposure in the last three months and with OCDS score. This finding suggests that the behavioral pattern of compulsive drinking is related to the concentration of brain iron in the dorsal striatum, a brain region involved in habituation and automated behaviors. The specificity of this finding is supported by the lack of correlation of momentary alcohol urges and severity of alcohol dependence with the striatal iron levels.

### Accumulation of brain iron in AUD

As hypothesized, increased accumulation of brain iron was observed in AUD participants. Alcohol use has a significant and wide-ranging impact on multi-systems/organs and might be associated with systemic iron accumulation in the body. Alcohol use may increase intestinal iron absorption and be related to abnormal hepcidin signaling (Duane et al. 1992; Juhás et al. 2017; Kohgo et al. 2008). The liver, as the major storage site for iron, as well as the principal targets for alcohol injury, suffers from iron overload (Ioannou et al. 2004; Tavill and Qadri 2004). Further, it is reported that alcohol use disrupts the blood-brain barrier (BBB) integrity (Haorah et al. 2005; Pimentel et al. 2020), which could have impact on iron transport and contributes to brain iron accumulation (Olmedo-Díaz et al. 2017). What is more, the pre-clinical experimental literature reports increased brain iron after acute and chronic alcohol exposure in animals (Crews and Nixon 2009; Rouach et al. 1997; Rouach et al. 1990), which was hypothesized to be related to free radicals and oxidative stress, and consequently in neuroinflammation. Studies in humans linked long-term alcohol use and AUD to signs of increased immune signaling in the central nervous system (Coller and Hutchinson 2012), pro-inflammatory state in the brain (Rubio-Araiz et al. 2017), and microglia activation (Kempuraj et al. 2016; Petrakis et al. 2019). However, the mechanism underlying increased brain iron in AUD and its relation to neuroinflammation and neurodegeneration is still not fully understood. (Haorah et al. 2005).

### Specific brain iron accumulation in the dorsal striatum

Our findings indicate a specific brain iron accumulation in the dorsal striatum of AUD participants. Whole-brain analysis showed significantly higher susceptibility in the dorsal striatum of AUD participants compared to healthy controls. A potential reason why striatal regions might be particularly sensitive to iron accumulation is its high energetic demands resulting from dopaminergic activity. In dopamine synthesis, iron is a co-factor of tyrosine hydroxylase, which converts tyrosine to dopamine. Tissue culture experiments in peripheral blood cells have shown that dopamine alters cellular iron homeostasis by increasing iron incorporation (Dichtl et al. 2018). The dorsal striatum is particular vulnerable to alterations of the iron homeostasis because it holds the highest density in dopaminergic terminals, and dopamine turnover and metabolism are energetically extremely demanding with iron and dopamine forming a potent redox couple (Hare and Double 2016; Scheurich et al. 2005), which might also underlie the higher sensitivity of the dorsal vs. ventral striatal regions to neurodegeneration in Parkinson’s disease. Following the dopamine synthesis, molecules from oxidation in the dopamine degradation could be neurotoxic to catecholaminergic cells (Muñoz et al. 2012), and iron was found as a mediator of the neurotoxicity in Parkinson’s disease via Fe − dopamine complex (Paris et al. 2005).

Thus, regions with high dopaminergic activity appear to be vulnerable to iron accumulation. This in turn might lead to cognitive and behavioral impairment (Rodrigue et al. 2020; Schröder et al. 2013; Spence et al. 2020; Tonekaboni and Mollamohammadi 2014). In fact, evidence from human PET and postmortem studies and corresponding animal experiments demonstrated profound alterations in the dopamine system in AUD (Hansson et al. 2019; Hirth et al. 2016). The ventral and dorsal striatum play different dopamine-mediated roles in addiction, and the dorsal striatum is more related to compulsive use. (Ito et al. 2000; Lüscher et al. 2020; Uhl et al. 2019; Vollstädt-Klein et al. 2010). In the present study, AUD participants had been drinking for 19.8 years, on average, and were therefore likely in the stage of compulsive use, to varying degrees as assessed by the OCDS. Correspondingly, the dopaminergic activity in the dorsal striatum might have become dominant in their alcohol use behavior, which led to increased iron accumulation in this region.

### Connections between brain iron accumulation and compulsive drinking in AUD

The current study found a positive correlation between dorsal striatal susceptibility, i.e., iron load, and compulsive drinking behavior as measured by the OCDS (Vollstädt-Klein et al. 2010). This correlation further strengthens the hypothesis that the dorsal striatum is specifically involved with mediating compulsive drinking behavior and that a potential underlying neural mechanism contributing to this might be iron overload (Tonekaboni and Mollamohammadi 2014). An interesting question these findings raise is whether brain iron accumulation in the dorsal striatum is a predisposing factor for compulsive behavior and the development of AUD or whether it is the result of long-term alcohol consumption. In order to explore this question it would be useful to follow individuals over trajectory of addiction development, to make a direct intra-individual comparison of iron levels over time. While the present study is limited by its cross-sectional design and found a positive correlation between brain iron accumulation and the drinking amount, some recent studies have attempted to elucidate the relationship between brain iron, cognitive function, and age in non-AUD populations. Interestingly, in healthy individuals, greater iron load was predictive of deficits in a working memory task, especially in younger and middle-aged participants, when compared to older ones (Rodrigue et al. 2020). However, a different study (Larsen et al. 2020) in which the longitudinal trajectories of striatal iron load were examined came to the conclusion that greater cognitive ability is increasingly associated with greater iron concentration through late adolescence and young adulthood. Meanwhile, we did not find significant correlations between dorsal striatal susceptibility and AUQ or ADS scores. We did not find this result surprising given that the AUQ assesses ‘state’ as opposed to ‘trait,’ which reflects a temporary condition and would be unlikely to correlate with a cumulative, chronic indicator like iron load. The ADS, on the other hand, does in fact measure trait (severity of alcohol dependence), but one which consists of several domains beyond compulsivity, including negative emotion, preoccupation and salience. Therefore, it seems likely that the ADS may associate with neural activity that goes beyond the dorsal striatum. The OCDS is a tool that is specific to the assessment of trait compulsive drinking and its positive correlation with dorsal striatal susceptibility makes a compelling case that increased iron load in the dorsal striatum is directly related to increased compulsive drinking patterns.

### Clinical perspective

These results provide us with a new perspective on clinical assessment and treatment. Brain iron concentration from imaging examinations could function as a potential biological marker in AUD diagnosis, providing an objective measure associated with recent alcohol exposure and compulsive drinking, which might be helpful for individualized treatment of AUD.

Importantly, it must be noted that increased brain iron accumulation leads to signal loss and hence systematic artefacts when acquiring fMRI images because of the static field inhomogeneities. This represents a specific challenge for clinical addiction researchers using fMRI, because it is exactly these regions—the putamen, pallidus, insula, and caudate—that have been hypothesized to have special relevance for the development and maintenance of addiction. Meanwhile, these regions are disproportionately affected by iron accumulation when compared to healthy individuals (Puckett et al. 2018; Song 2001). This likely has significant implications when analyzing fMRI data and should be regarded as a potentially impacting factor in studies of AUD.

### Limitation

Our re-analyses following this innovative method included existing datasets from previous projects using the same inclusion criteria and scanning parameters. This resulted in limitations regarding data resolution. Second, although our GRE sequence appears suitable for standard QSM methods (Haacke et al. 2015), its spatial resolution is relatively low, which may have limited our ability to detect iron increases in smaller brain regions of the mid and hind brain as previously reported (Juhás et al. 2017; Topiwala et al. 2022a) and prevented the exploration of striatal subregions. However, this work performed whole-brain analysis and warrants further investigation using QSM of adequate spatial resolution. Moreover, our analyses only found a significant correlation in both groups, but not within the AUD group alone. This might be because of the classification based on DSM criteria, which results in different distributions in the AUD and healthy individuals. Third, we had no access to blood markers (e.g., iron levels, ferritin, and transferrin saturation), and were unable to study the relationship between iron metabolism and brain iron accumulation. Thus, future studies need to address these issues by using state-of-the-art sequences, including biomarkers of peripheral iron metabolism, and most importantly by positing an a priori and pre-registered hypothesis on the effect of iron accumulation on behavioral and other clinical outcomes.

## Conclusion

This is the first study exploring whole-brain iron accumulation in AUD using GRE sequences with a large clinical sample. It is also the first time that compulsive behavioral patterns in AUD have been related to brain iron accumulation. In summary, treating compulsive patterns of alcohol use is one of the main aims in clinical practice with regard to AUD. The neural mechanisms underlying habituation and compulsivity are still not fully understood. This study using QSM susceptibility measures finds increased iron accumulation in the dorsal striatum to be associated with the behavior of compulsive drinking, which might bring a new perspective to clinical practice. Further, neuroinflammation might be a consequence of brain iron accumulation which might relate AUD to neuroinflammation mechanisms. Lastly, our results also have implications for fMRI methods used in addiction research, because iron accumulation results in signal dropout when echo planar imaging images are acquired. This means that regions of the basal ganglia, specifically of interest in general SUD research, have a potentially systematically disturbed signal, which may affect the quality of the analysis. The method used in the current study is easy to implement and offers the possibility to examine brain iron accumulation with images using short GRE sequences, which might already have been acquired in previous studies as images for fieldmap correction.

## Supplementary Information

ESM 1 (PDF 270 kb)

## References

[CR1] American Psychiatric Association (2013). Diagnostic and statistical manual of mental disorders (DSM-5®).

[CR2] Anton RF, Moak DH, Latham P (1995). The Obsessive Compulsive Drinking Scale: a self-rated instrument for the quantification of thoughts about alcohol and drinking behavior. Alcoholism.

[CR3] Bach P, Hill H, Reinhard I, Gädeke T, Kiefer F, Leménager T (2021) Reliability of the fMRI-based assessment of self-evaluation in individuals with internet gaming disorder. Eur Arch Psychiatry Clin Neurosci 10.1007/s00406-021-01307-2 PMC9388403 34275007

[CR4] Bach P, Zois E, Vollstädt-Klein S, Kirsch M, Hoffmann S, Jorde A, Frank J, Charlet K, Treutlein J, Beck A, Heinz A, Walter H, Rietschel M, Kiefer F (2019). Association of the alcohol dehydrogenase gene polymorphism rs1789891 with gray matter brain volume, alcohol consumption, alcohol craving and relapse risk. Addict Biol.

[CR5] Ben-Shachar D, Livne E, Spanier I, Zuk R, Youdim MB (1993). Iron modulates neuroleptic-induced effects related to the dopaminergic system. Isr J Med Sci.

[CR6] Ben-Shachar D, Youdim MB (1990). Neuroleptic-induced supersensitivity and brain iron: I. Iron deficiency and neuroleptic-induced dopamine D2 receptor supersensitivity. J Neurochem.

[CR7] Bohn MJ, Krahn DD, Staehler BA (1995). Development and initial validation of a measure of drinking urges in abstinent alcoholics. Alcohol Clin Exp Res.

[CR8] Coller JK, Hutchinson MR (2012). Implications of central immune signaling caused by drugs of abuse: mechanisms, mediators and new therapeutic approaches for prediction and treatment of drug dependence. Pharmacol Ther.

[CR9] Crews FT, Nixon K (2009). Mechanisms of neurodegeneration and regeneration in alcoholism. Alcohol Alcohol.

[CR10] de Rochefort L, Liu T, Kressler B, Liu J, Spincemaille P, Lebon V, Wu J, Wang Y (2010). Quantitative susceptibility map reconstruction from MR phase data using Bayesian regularization: validation and application to brain imaging. Magn Reson Med.

[CR11] De Santis S, Bach P, Pérez-Cervera L, Cosa-Linan A, Weil G, Vollstädt-Klein S, Hermann D, Kiefer F, Kirsch P, Ciccocioppo R (2019). Microstructural white matter alterations in men with alcohol use disorder and rats with excessive alcohol consumption during early abstinence. JAMA Psychiat.

[CR12] Deistung A, Schweser F, Reichenbach JR (2017). Overview of quantitative susceptibility mapping. NMR Biomed.

[CR13] Dichtl S, Haschka D, Nairz M, Seifert M, Volani C, Lutz O, Weiss G (2018). Dopamine promotes cellular iron accumulation and oxidative stress responses in macrophages. Biochem Pharmacol.

[CR14] Duane P, Raja KB, Simpson RJ, Peters TJ (1992). Intestinal iron absorption in chronic alcoholics. Alcohol Alcohol.

[CR15] Ersche KD, Acosta-Cabronero J, Jones PS, Ziauddeen H, van Swelm RP, Laarakkers CM, Raha-Chowdhury R, Williams GB (2017). Disrupted iron regulation in the brain and periphery in cocaine addiction. Transl Psychiatry.

[CR16] Fritz M, Klawonn AM, Zahr NM (2022). Neuroimaging in alcohol use disorder: from mouse to man. J Neurosci Res.

[CR17] Gerchen MF, Weiss F, Kirsch M, Rentsch A, Halli P, Kiefer F, Kirsch P (2021). Dynamic frontostriatal functional peak connectivity (in alcohol use disorder). Hum Brain Mapp.

[CR18] Haacke EM, Liu S, Buch S, Zheng W, Wu D, Ye Y (2015). Quantitative susceptibility mapping: current status and future directions. Magn Reson Imaging.

[CR19] Hansson AC, Gründer G, Hirth N, Noori HR, Spanagel R, Sommer WH (2019). Dopamine and opioid systems adaptation in alcoholism revisited: convergent evidence from positron emission tomography and postmortem studies. Neurosci Biobehav Rev.

[CR20] Hansson AC, Koopmann A, Uhrig S, Bühler S, Domi E, Kiessling E, Ciccocioppo R, Froemke RC, Grinevich V, Kiefer F, Sommer WH, Vollstädt-Klein S, Spanagel R (2018) Oxytocin reduces alcohol cue-reactivity in alcohol-dependent rats and humans. Neuropsychopharmacology 43: 1235-1246. 10.1038/npp.2017.257 PMC5916348 29090683

[CR21] Haorah J, Heilman D, Knipe B, Chrastil J, Leibhart J, Ghorpade A, Miller DW, Persidsky Y (2005). Ethanol-induced activation of myosin light chain kinase leads to dysfunction of tight junctions and blood-brain barrier compromise. Alcoholism.

[CR22] Hare DJ, Double KL (2016). Iron and dopamine: a toxic couple. Brain.

[CR23] Hirth N, Meinhardt MW, Noori HR, Salgado H, Torres-Ramirez O, Uhrig S, Broccoli L, Vengeliene V, Roßmanith M, Perreau-Lenz S, Köhr G, Sommer WH, Spanagel R, Hansson AC (2016). Convergent evidence from alcohol-dependent humans and rats for a hyperdopaminergic state in protracted abstinence. Proc Natl Acad Sci.

[CR24] Hubertus S, Thomas S, Cho J, Zhang S, Wang Y, Schad LR (2019). Comparison of gradient echo and gradient echo sampling of spin echo sequence for the quantification of the oxygen extraction fraction from a combined quantitative susceptibility mapping and quantitative BOLD (QSM + qBOLD) approach. Magn Reson Med.

[CR25] Hubertus S, Thomas S, Cho J, Zhang S, Wang Y, Schad LR (2019). Using an artificial neural network for fast mapping of the oxygen extraction fraction with combined QSM and quantitative BOLD. Magn Reson Med.

[CR26] Ioannou GN, Dominitz JA, Weiss NS, Heagerty PJ, Kowdley KV (2004). The effect of alcohol consumption on the prevalence of iron overload, iron deficiency, and iron deficiency anemia. Gastroenterology.

[CR27] Ito R, Dalley JW, Howes SR, Robbins TW, Everitt BJ (2000). Dissociation in conditioned dopamine release in the nucleus accumbens core and shell in response to cocaine cues and during cocaine-seeking behavior in rats. J Neurosci.

[CR28] Ito R, Dalley JW, Robbins TW, Everitt BJ (2002). Dopamine release in the dorsal striatum during cocaine-seeking behavior under the control of a drug-associated cue. J Neurosci.

[CR29] Juhás M, Sun H, Brown MRG, MacKay MB, Mann KF, Sommer WH, Wilman AH, Dursun SM, Greenshaw AJ (2017). Deep grey matter iron accumulation in alcohol use disorder. NeuroImage.

[CR30] Karl D, Bumb JM, Bach P, Dinter C, Koopmann A, Hermann D, Mann K, Kiefer F, Vollstädt-Klein S (2021) Nalmefene attenuates neural alcohol cue-reactivity in the ventral striatum and subjective alcohol craving in patients with alcohol use disorder. Psychopharmacology. 10.1007/s00213-021-05842-7 PMC8292278 33846866

[CR31] Kempuraj D, Thangavel R, Natteru PA, Selvakumar GP, Saeed D, Zahoor H, Zaheer S, Iyer SS, Zaheer A (2016). Neuroinflammation induces neurodegeneration. J Neurol Neurosurg Spine.

[CR32] Kivlahan DR, Sher KJ, Donovan DM (1989). The Alcohol Dependence Scale: a validation study among inpatient alcoholics. J Stud Alcohol.

[CR33] Kohgo Y, Ohtake T, Ikuta K, Suzuki Y, Torimoto Y, Kato J (2008). Dysregulation of systemic iron metabolism in alcoholic liver diseases. J Gastroenterol Hepatol.

[CR35] Kurz FT, Buschle LR, Rotkopf LT, Herzog FS, Sterzik A, Schlemmer HP, Kampf T, Bendszus M, Heiland S, Ziener CH (2021) Dependence of the frequency distribution around a sphere on the voxel orientation. Z Med Phys 10.1016/j.zemedi.2021.01.005 33750628

[CR36] Langkammer C, Schweser F, Krebs N, Deistung A, Goessler W, Scheurer E, Sommer K, Reishofer G, Yen K, Fazekas F (2012). Quantitative susceptibility mapping (QSM) as a means to measure brain iron? A post mortem validation study. Neuroimage.

[CR37] Larsen B, Bourque J, Moore TM, Adebimpe A, Calkins ME, Elliott MA, Gur RC, Gur RE, Moberg PJ, Roalf DR, Ruparel K, Turetsky BI, Vandekar SN, Wolf DH, Shinohara RT, Satterthwaite TD (2020). Longitudinal development of brain iron is linked to cognition in youth. J Neurosci.

[CR38] Liu T, Liu J, De Rochefort L, Spincemaille P, Khalidov I, Ledoux JR, Wang Y (2011). Morphology enabled dipole inversion (MEDI) from a single-angle acquisition: comparison with COSMOS in human brain imaging. Magn Reson Med.

[CR39] Lüscher C, Robbins TW, Everitt BJ (2020). The transition to compulsion in addiction. Nat Rev Neurosci.

[CR40] Mann K, Ackermann K (2000). Die OCDS-G: Psychometrische Kennwerte der deutschen Version der obsessive compulsive drinking scale. Sucht.

[CR41] Melega WP, Laćan G, Harvey DC, Way BM (2007). Methamphetamine increases basal ganglia iron to levels observed in aging. Neuroreport.

[CR42] Möller HE, Bossoni L, Connor JR, Crichton RR, Does MD, Ward RJ, Zecca L, Zucca FA, Ronen I (2019). Iron, myelin, and the brain: neuroimaging meets neurobiology. Trends Neurosci.

[CR43] Muñoz P, Huenchuguala S, Paris I, Segura-Aguilar J (2012). Dopamine oxidation and autophagy. Parkinson’s Disease.

[CR44] Necus J, Smith FE, Thelwall PE, Flowers CJ, Sinha N, Taylor PN, Blamire AM, Wang Y, Cousins DA (2019). Quantification of brain proton longitudinal relaxation (T(1) ) in lithium-treated and lithium-naïve patients with bipolar disorder in comparison to healthy controls. Bipolar Disord.

[CR45] Olmedo-Díaz S, Estévez-Silva H, Orädd G, Af Bjerkén S, Marcellino D, Virel A (2017). An altered blood–brain barrier contributes to brain iron accumulation and neuroinflammation in the 6-OHDA rat model of Parkinson’s disease. Neuroscience.

[CR46] Paris I, Martinez-Alvarado P, Cárdenas S, Perez-Pastene C, Graumann R, Fuentes P, Olea-Azar C, Caviedes P, Segura-Aguilar J (2005). Dopamine-dependent iron toxicity in cells derived from rat hypothalamus. Chem Res Toxicol.

[CR47] Petrakis IL, Ralevski E, Gueorguieva R, Sloan ME, Devine L, Yoon G, Arias AJ, Sofuoglu M (2019). Targeting neuroinflammation with minocycline in heavy drinkers. Psychopharmacology.

[CR48] Pimentel E, Sivalingam K, Doke M, Samikkannu T (2020) Effects of drugs of abuse on the blood-brain barrier: a brief overview. Front Neurosci 14 10.3389/fnins.2020.00513 PMC7326150 32670001

[CR49] Puckett AM, Bollmann S, Poser BA, Palmer J, Barth M, Cunnington R (2018). Using multi-echo simultaneous multi-slice (SMS) EPI to improve functional MRI of the subcortical nuclei of the basal ganglia at ultra-high field (7 T). NeuroImage.

[CR50] Rodrigue KM, Daugherty AM, Foster CM, Kennedy KM (2020). Striatal iron content is linked to reduced fronto-striatal brain function under working memory load. NeuroImage.

[CR51] Rouach H, Houzé P, Gentil M, Orfanelli M-T, Nordmann R (1997). Changes in some pro-and antioxidants in rat cerebellum after chronic alcohol intake. Biochem Pharmacol.

[CR52] Rouach H, Houze P, Orfanelli M-T, Gentil M, Bourdon R, Nordmann R (1990). Effect of acute ethanol administration on the subcellular distribution of iron in rat liver and cerebellum. Biochem Pharmacol.

[CR53] Rubio-Araiz A, Porcu F, Pérez-Hernández M, García-Gutiérrez MS, Aracil-Fernández MA, Gutierrez-López MD, Guerri C, Manzanares J, O'Shea E, Colado MI (2017). Disruption of blood–brain barrier integrity in postmortem alcoholic brain: preclinical evidence of TLR4 involvement from a binge-like drinking model. Addict Biol.

[CR54] Scheurich A, Müller MJ, Anghelescu I, Lörch B, Dreher M, Hautzinger M, Szegedi A (2005). Reliability and validity of the form 90 interview. Eur Addict Res.

[CR55] Schröder N, Figueiredo LS, de Lima MN (2013). Role of brain iron accumulation in cognitive dysfunction: evidence from animal models and human studies. J Alzheimer's dis.

[CR56] Sjoerds Z, de Wit S, van den Brink W, Robbins TW, Beekman ATF, Penninx BWJH, Veltman DJ (2013). Behavioral and neuroimaging evidence for overreliance on habit learning in alcohol-dependent patients. Transl Psychiatry.

[CR57] Song AW (2001). Single-shot EPI with signal recovery from the susceptibility-induced losses. Magn Reson Med.

[CR58] Spence H, McNeil CJ, Waiter GD (2020). The impact of brain iron accumulation on cognition: a systematic review. PLoS One.

[CR59] Sun H, Wilman AH (2014). Background field removal using spherical mean value filtering and Tikhonov regularization. Magn Reson Med.

[CR60] Tavill AS, Qadri AM (2004). Alcohol and iron. Semin Liver Dis.

[CR61] Tonekaboni SH, Mollamohammadi M (2014). Neurodegeneration with brain iron accumulation: an overview. Iran J Child Neurol.

[CR62] Topiwala A, Wang C, Ebmeier KP, Burgess S, Bell S, Levey DF, Zhou H, McCracken C, Roca-Fernandez A, Petersen S (2022a) Impact of moderate alcohol consumption on brain iron and cognition: observational and genetic analyses. 10.1371/journal.pmed.1004039 PMC9282660 35834561

[CR63] Topiwala A, Wang C, Ebmeier KP, Burgess S, Bell S, Levey DF, Zhou H, McCracken C, Roca-Fernández A, Petersen SE, Raman B, Husain M, Gelernter J, Miller KL, Smith SM, Nichols TE (2022). Associations between moderate alcohol consumption, brain iron, and cognition in UK Biobank participants: observational and mendelian randomization analyses. PLoS Med.

[CR64] Uhl GR, Koob GF, Cable J (2019). The neurobiology of addiction. Ann N Y Acad Sci.

[CR65] Vanderschuren LJMJ, Di Ciano P, Everitt BJ (2005). Involvement of the dorsal striatum in cue-controlled cocaine seeking. J Neurosci.

[CR66] Vollstädt-Klein S, Gerhardt S, Lee A, Strosche A, Sharafi G, Nuriyeva R, Seidt J, Hennig O, Alm B, Hermann D, Sommer WH, Kiefer F, Luderer M, Sobanski E (2020). Interaction between behavioral inhibition and neural alcohol cue-reactivity in ADHD and alcohol use disorder. Psychopharmacology.

[CR67] Vollstädt-Klein S, Wichert S, Rabinstein J, Bühler M, Klein O, Ende G, Hermann D, Mann K (2010). Initial, habitual and compulsive alcohol use is characterized by a shift of cue processing from ventral to dorsal striatum. Addiction.

[CR68] Wang Y, Liu T (2015). Quantitative susceptibility mapping (QSM): Decoding MRI data for a tissue magnetic biomarker. Magn Reson Med.

[CR69] Whitfield JB, Zhu G, Heath AC, Powell LW, Martin NG (2001). Effects of alcohol consumption on indices of iron stores and of iron stores on alcohol intake markers. Alcohol Clin Exp Res.

[CR70] Yao S, Zhong Y, Xu Y, Qin J, Zhang N, Zhu X, Li Y (2017). Quantitative susceptibility mapping reveals an association between brain iron load and depression severity. Front Hum Neurosci.

[CR71] Zhou D, Liu T, Spincemaille P, Wang Y (2014). Background field removal by solving the Laplacian boundary value problem. NMR Biomed.

