# Supplementary material for: Association between iron accumulation in the dorsal striatum and compulsive drinking in alcohol use disorder
Source: Psychopharmacology (Berl). 2022 Dec 29;240(2):249–57. doi: 10.1007/s00213-022-06301-7 (PMC9879829; doi:10.1007/s00213-022-06301-7)
Supplement: Supplementary file 1 — (PDF 270 kb) [file 213_2022_6301_MOESM1_ESM.pdf]

Supplementary Material

Study name	Inclusion criterion *	Sample size		ClinicalTrials/ German Clinical Trials Register (DRKS)
		AUD	HC	
Transalc [22]	1) Alcohol Use Disorder according to DSM-5;<br>2) for heavy-drinker, at least 84g pure alcohol in the previous 90 day;<br>3) treatment-seeking and abstinent.	33	37	DRKS: DRKS00003357
SFB_Folgestudie [23]	1) alcohol dependence according to the DSM-IV and ICD 10;<br>3) treatment-seeking and abstinent.	82	53	DRKS: DRKS00003388
ADHS [24]	1) Alcohol Use Disorder according to the DSM-5;<br>2) treatment-seeking and abstinent.	21	20	DRKS: DRKS00004929
eMEDs [25]	1) alcohol dependence according to the DSM-IV and ICD 10;<br>2) treatment-seeking and abstinent.	30	58	DRKS: DRKS00003341
NALCUE [26]	1) Alcohol Use Disorder according to the DSM-5;<br>2) at least 60g for men and 40g for women pure alcohol, at least 5 days/week;<br>3) non-treatment-seeking.	20	1	ClinicalTrials: NCT02372318
Oxytocin [27]	healthy participants **	0	13	DRKS: DRKS00009253
Avatar [28]	healthy participants	0	49	DRKS: DRKS00009439
CBD-IS (not yet published)	healthy participants	0	43	ClinicalTrials: NCT02051387

Abbreviations: diagnostic and statistical manual of mental disorders (DSM), international classification of diseases (ICD), Alcohol Use Disorder (AUD), healthy control (HC).

* General criterion of inclusion and exclusion:
These studies included AUD individuals who 1) between 18 and 75 years, 2) right-handed; and excluded AUD individuals who 1) comorbid axis-I disorders (other than nicotine dependence) in the last year, 2) treatment with psychotropic or anticonvulsive medications in the last three months, 3) severe neurological or physiological disease (i.e. liver cirrhosis), 4) positive drug screening, 5) ineligibility for MRI scanning (e.g. metal implants), 6) history of severe head trauma.

These studies included healthy participants/light to moderate drinkers who 1) were aged between 18 and 75 years, 2) right-handed, 3) had an average alcohol consumption below 14g pure alcohol; and excluded healthy participants who 1) comorbid axis-I disorders (other than nicotine dependence) in the last year, 2) treatment with psychotropic or anticonvulsive medications in the last three months, 3) severe neurological or physiological disease (i.e. liver cirrhosis), 4) positive drug screening, 5) ineligibility for MRI scanning (e.g. metal implants), 6) history of severe head trauma.[27]

** light to moderate drinkers were classified as healthy participants.

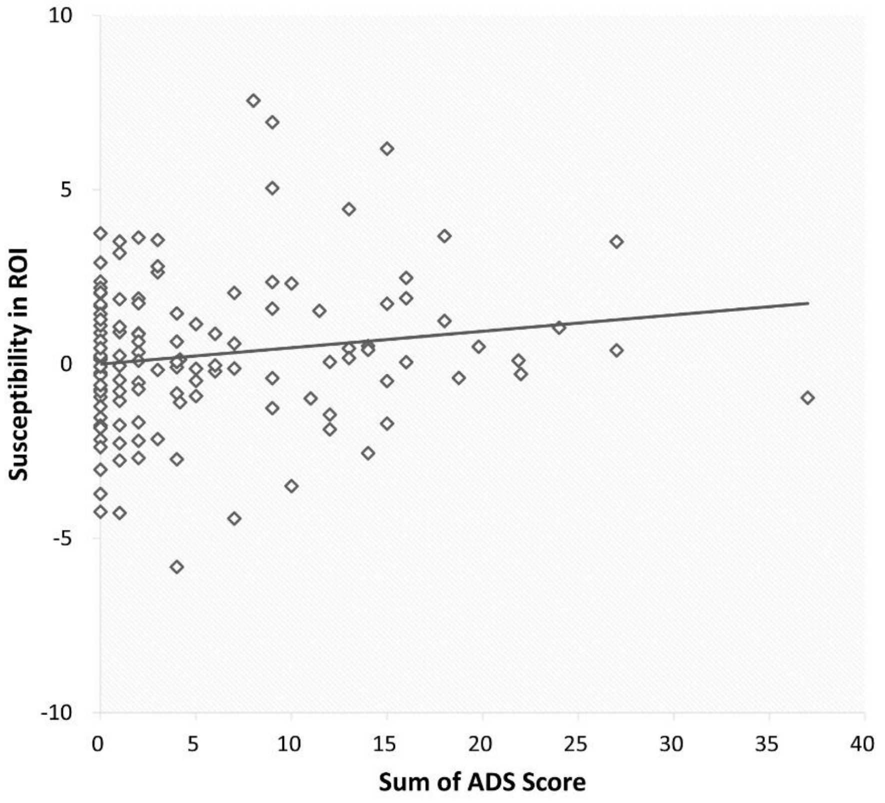

sFigure 1. Correlation of sum of ADS scores and susceptibility, controlling age and smoke-status

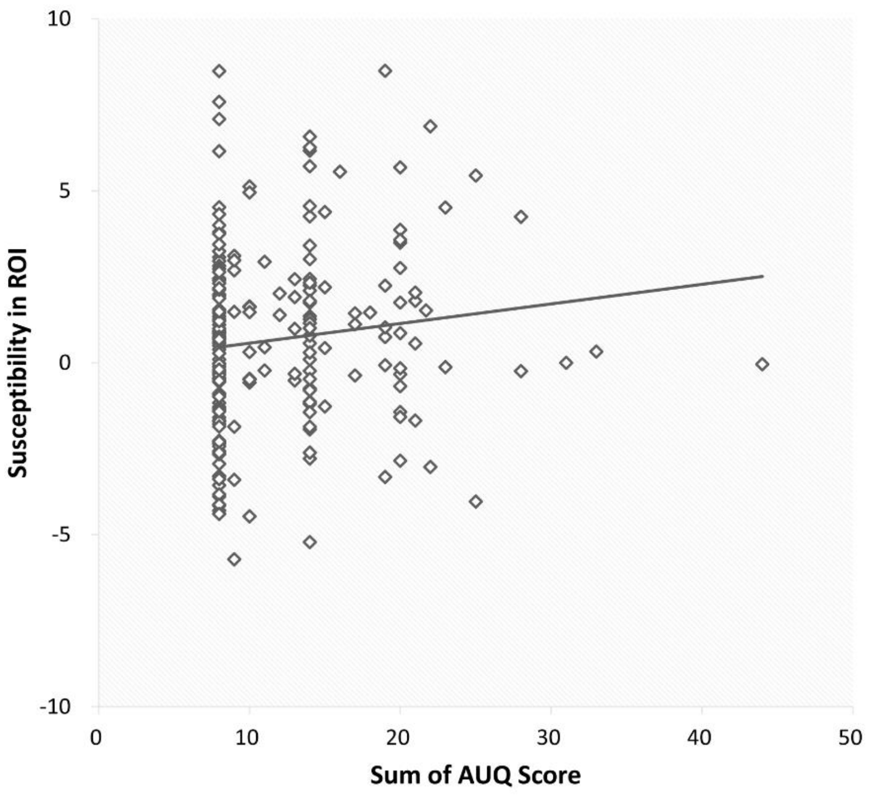

sFigure 2. Correlation of sum of AUQ scores and susceptibility, controlling age and smoke-status
